# Scaling up Business Plans in Tajikistan: a qualitative study of the history, barriers, facilitators and lessons learnt

**DOI:** 10.1080/16549716.2021.1947552

**Published:** 2021-08-03

**Authors:** Sarah S Werner, Gulara Afandiyeva, Gulzira Karimova, Sabine Kiefer, Nasrullo Abdujabborov, Muazamma Dzhamalova, Ilhom Bandaev, Helen Prytherch

**Affiliations:** a University College Freiburg, Albert-Ludwigs-University Freiburg, Freiburg, Germany; b “Enhancing Primary Health Care Services” Project, Representative Office of the Swiss Tropical and Public Health Institute, Dushanbe, Tajikistan; c Swiss Center for International Health, Swiss Tropical and Public Health Institute, Basel, Switzerland; d University of Basel, Basel, Switzerland; e Swiss Cooperation Office, Swiss Agency for Development and Cooperation, Dushanbe, Tajikistan; f Ministry of Health and Social Protection of Population, Dushanbe, Republic of Tajikistan

**Keywords:** Business Planning, vertical scale-up, horizontal scale-up, health planning, ExpandNet/WHO

## Abstract

**Background:**

To improve health planning at primary health care (PHC) level, Business Plans were introduced in Tajikistan by the Enhancing Primary Health Care (EPHC) Services Project.

**Objective:**

To describe the history and process of implementation of Business Plans and to identify barriers, facilitators and lessons learnt from scaling up Business Plans.

**Methods:**

Set in a qualitative research design, we conducted a desk review of project and official documents and seventeen semi-structured interviews with key stakeholders at national and sub-national levels between May and July 2020. We used an interview guide informed by the ExpandNet/WHO framework and analyzed the data following a content analysis approach facilitated by MAXQDA.

**Results:**

With the participation of various user organizations and resource teams and through a variety of strategic scale-up choices, Business Plans have been scaled up from a vertical pilot project to institutionalized health management tools covering 45% of Tajikistan’s PHC facilities. The most prominent facilitators for scaling up Business Plans were the institutionalization and integration of the tool into the Tajik health system, the close collaboration with Community Health Teams (CHTs), the high acceptance of the tool among the users, the advocacy through champions and policy-makers and the large dissemination network. The most outstanding barriers to scaling up Business Plans were insufficient financial or human resources, general weaknesses in health governance, the lack of a strategic scale-up plan and strategic decisions, the lack of motivation or overall vision to implement Business Plans at a large scale and difficulties in donor coordination.

**Conclusion:**

To ensure the continuity of scaling up Business Plans, developing a scale-up strategy, strengthening cross-sectoral collaboration and participation during scaling up, and capacitating the user organizations of Business Plans are important next steps to ensure the sustainability and effectiveness of Business Plans in the future.

## Background

In recent years, strategies to improve health systems have focused on Primary Health Care (PHC) services. PHC, also referred to as family medicine, is considered to be an effective and efficient approach to improving access to healthcare services, health system performance and population health [1–3]. From the early stages onwards, weak management, particularly at district level, is considered to be a major barrier to construct well-performing PHC systems [4–6].

This paper focuses on scaling up Business Plans (health facility management tools) in Tajikistan. Following the collapse of the Soviet Union, Tajikistan has undergone a health sector reform, shifting the focus from specialty care to primary health care [7]. In order to facilitate decentralized health planning at district level, a set of Business Planning tools (from now on referred to as Business Plans) was designed by the Enhancing Primary Health Care (EPHC) Services Project to improve the managerial capacities and the autonomy of health care providers. The Business Plans proved to be effective in improving transparency regarding the allocation and subsequent utilization of funds and the management of health services, as well as the responsiveness of health care managers to population health needs, resulting in an improvement of health outcomes at the PHC level [8].

Based on their success in the pilot phase, the decision was taken to scale up the Business Plans. There are nuanced differences between the terms ‘scaling’, ‘scale-up’ or ‘scaling up’ [9], and the term ‘scaling up’ is often used to refer to the“spread” [10, p.2] or “expanding the coverage of health interventions” [11,p.85]. Here we use the term ‘scaling up’ which is defined by the World Health Organization (WHO) as “deliberate efforts to increase the impact of successfully tested health innovations so as to benefit more people and to foster policy and programs development on a lasting basis“ [12,p.2]. Successfully scaling up health interventions can expand the access and reach of health care to other target populations [10,13]. However, expanding coverage of public health interventions, especially when integrating vertical programs into the existing health system [14], often faces a complex array of barriers [11,15]. Despite widespread agreement on the importance of scaling up health interventions or other health-related practices to improve health systems, most research on scaling up has focused on the intervention itself, rather than assessing the decisive processes, strategies and actors involved in successfully scaling up health services [9,12,16,17]. Scholars have emphasized that more empirical studies are needed to identify the determinants of successful scaling up attempts [18]. However, implementation research – the “scientific study of methods to promote the systematic uptake of research findings and other evidence-based practices into routine practice” [19,p.1] – has so far received little attention and priority, and consequently also little funding [20].

The objectives of this study are (a) to describe the history, process of implementation and consolidation of Business Plans in the Tajik health system by means of the ExpandNet/WHO framework, (b) to identify barriers and facilitators to scaling up, and based on that (c) to extract lessons learnt related to scaling up health innovations.

## Methods

### Study design

**Figure 1.**
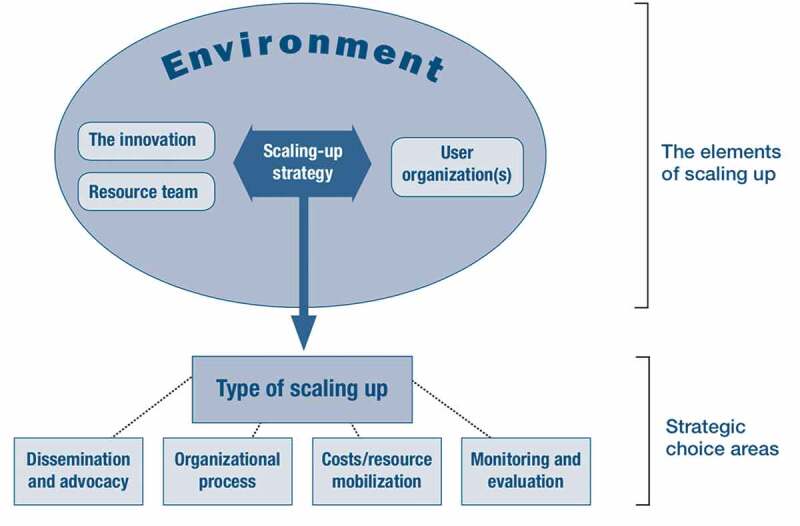
The ExpandNet/WHO Framework [22]. Reproduced with permission from the World Health Organization

We performed a qualitative research design to assess the scale-up process and management of Business Plans in PHC facilities in Tajikistan by implementing two steps: (1) a desk review and (2) semi-structured interviews analyzed using content analysis. There are several frameworks to describe scaling up health interventions [10,12,15,21–24]. We chose to follow the ExpandNet/WHO framework as it is based on experience in low- and middle-income countries, among others, on processes of scaling up PHC pilot projects. Due to its breadth of conceptual dimensions and the detailed guidance it offers for a systematic analysis of scaling up, we preferred it over other analytical frameworks for understanding scale-up processes, such as those developed by Hanson et al. [15] or Yamey [24]. As depicted in Figure 1, the ExpandNet/WHO framework conceptualizes scaling up into five elements: (1) the innovation (an intervention that is new to its context and is being scaled up), (2) the resource team(s) (the individuals, institutions or organizations that support scaling up an innovation), (3) the user organization(s) (the individuals, institutions or organizations that are expected to implement and scale up the innovation), (4) the scaling up strategy (the actions, steps or plans needed to integrate the innovation into policies and programs) and (5) the environment (external conditions or institutions that have an impact on an innovation during its scale-up process) [21]. The framework also identifies five strategic choice areas: The type of scaling up (guided or spontaneous), dissemination and advocacy, organizational processes, cost/resource mobilization and monitoring and evaluation [12,21]. Guided scaling up is further distinguished into horizontal scaling up, which refers to the expansion of an innovation to new areas or populations, and vertical scaling up, which refers to the institutionalization of the innovation in national and sub-national regulatory systems and structures [12]. We followed the COREQ checklist for reporting the study design and data analysis [25].

### Participants

In total, seventeen key stakeholders at national and sub-national level participated in the interviews. The interviewees were purposively selected based on their expertise and acquaintance with Business Plans and were contacted via email and telephone by the authors and the EPHC Services Project manager at that time. None of the contacted persons refused to participate. The interviewees held leading positions in the EPHC Services Project (n = 6), departments or institutions in the Ministry of Health and Social Protection of the Population of the Republic of Tajikistan (MoHSP) (n = 6), the regional Business Plan working groups (n = 1), district level PHC management teams (n = 2) and donor agencies (n = 2). All interviewees received information about the scope and the aim of the study prior to participating. Ethical clearance was obtained from the MoHSP and informed consent was obtained from each participant.

### Setting

We collected the interview data between May and July 2020. The interviews were conducted via audio calls in settings allowing for privacy, with most study participants located either at home or at their workplace. To ensure confidentiality, no demographic data such as gender and age, is revealed in the findings.

### Data collection

First, we conducted a desk review of EPHC Service Project sources, such as reports, reviews, publications and technical documents, and of official government and policy documents, such as orders, concerning Business Plans. Second, the interview questions were developed by SSW based on the desk review, the ExpandNet/WHO framework (under special consideration of the document ‘20 Questions for Developing a Scaling Up Case Study’ [17]), and literature and case studies on scaling up public health innovations. The interview guide was pilot tested with the EPHC Services Project manager and the subsequent first three interviews were conducted jointly (SSW, EPHC Services Project staff member in Tajikistan) to allow for observation, feedback and clarification between the investigators. Then, five interviews were conducted in English (SSW) and nine in Tajik or Russian (EPHC Services Project staff member in Tajikistan) using the same interview guide in the respective language.

The duration of the interviews ranged between 25 minutes and 85 minutes. All interviews were recorded. No repeat interviews were carried out and the transcripts were not returned to the participants to receive feedback. After conducting the interviews, the material was perceived to be saturated as no new information emerged during the final interviews.

### Data analysis

A qualitative content analysis approach was used to analyze the interview data [26]. After transcription – and if applicable, translation – interviews were coded (SSW) using MAXQDA according to an a-priori coding scheme derived from the ExpandNet/WHO framework. An inductive approach was used to cover emergent categories. After data analysis, the results were presented to the research team, selected interviewees and relevant stakeholders to obtain feedback. Direct quotes are used to reflect categories and themes emerging in the data and participants’ verbatim statements which are linked to the corresponding transcript letter and their associated institution or profession.

## Results

### History and implementation of Business Plans

#### Context of Business Plans

Business Plans were first introduced in Tajikistan in 2005 as a donor-funded pilot study in the midst of the healthcare reforms, which sought to improve access to health care services by restructuring healthcare funding and developing an integrated PHC model based on family medicine. Several national policies and laws fostered a supportive environment for scaling up Business Plans (see Table 1). With these laws and policies on family medicine, the Tajik Government redefined the priorities of the health sector and set goals for the improvement of health care services, which purposely align with the goals and ideas behind the Business Plans.

**Table 1.** The elements of scaling up Business Plans [12,21]

Name and definition	Assessment of the element of scaling up Business Plans
**The innovation** ‘*a set of health service interventions that is being scaled up’* [21,p.7]	Business Plan tool set:
	Rural Health Centre (RHC) Business Plans: Four key areas focusing on status quo and planning, targets and actions, monitoring and analysis. Consolidated Business Plan: Summary of information regarding the PHC managers’ targets and actions, the targets of the health facilities (each RHC), and the results from on-site monitoring visits of the health facilities. Business Plan management trainings: Workshops consisting of training modules for regional working groups on Business Plans and trainings for PHC management teams and heads of health facilities.
	During scaling up, the Business Plan was diversified and more components were added to the initial Business Plan package:
	In 2014: Guideline on the involvement of Community Health Teams (CHTs) to increase the collaboration between health facilities and the communities they serve. In 2018: Introduction of a management training module in the Postgraduate Medical Institute (PGMI).
**The user organization** ‘*the institutions or organizations that seek or are expected to adopt and implement the innovation on a large scale’* [21,p.8]	Health policy level: Business Planning department in the Republican Center for Family Medicine (RCCFM)
	Team of 4 specialists In charge of the implementation and control of Business Plans Tasked with the national roll-out/expansion of Business Plans
	Regional level: Regional working groups on Business Plans
	Teams of 6 to 8 specialists In charge of training the PHC management teams and the heads of RHCs Responsible for supervising and coordinating the process of Business Plan implementation
	District level: PHC management team
	Responsible for initiating, monitoring and analyzing the Business Plans
	Facility level: Head doctors of PHC facilities
	Tasked with elaborating the Business Plan for their RHC and its affiliated Health Houses
	Community level: Representatives of CHTs
	Tasked with representing the wider community in all matters regarding Business Plans
**The resource team** *‘individuals and organizations that seek to promote and facilitate wider use of the innovation’* [12,p.6]	From 2005–2015: “Enhancing Primary Health Care Services” (EPHC) Project by the Swiss Centre for International Health and its representative office in Tajikistan Tasks: Provision of technical, managerial and policy expertise, financial support, Business Plan trainings, and monitoring and evaluation of the Business Plan implementation Since 2015: Supervision, oversight and financial support for the implementation of Business Plans 2015-present: Business Planning department in the RCCFM *Note*: The RCCFM acts both as a resource team and user organization for scaling up Business Plans. Tasks: Responsible for trainings, monitoring and evaluation of the Business Plan, scale Business Plans nationwide by introducing them in all districts Establishment of inter-governmental partnerships: Department of Health Reform, PHC and International Relations (ideational support and advocacy, especially in the course of vertical scale-up efforts) Republican Healthy Lifestyle Centre: Coordination of the cooperation between the resource teams and the CHTs.
**The environment** *‘conditions and institutions which are external to the user organization but fundamentally affect the prospect for scaling up’* [12,p.6]	Policies and laws:
	The Conception of Health Sector Reform in Tajikistan (2002) Law on family medicine (Order No. 676, 29 December 2010) The National Programme on the Development of Family Medicine 2011–2015 in Tajikistan The Strategy Plan of the Primary Health Care based on the Family Medicine Principles in the Republic of Tajikistan for 2016–2020 The National Health Strategy 2010–2020 and 2021–2030

#### Elements of scaling up Business Plans

The Business Plan, the innovation that is being scaled, is a set of tools administered by health facilities and PHC management teams. At the health facility levels, head doctors elaborate a Rural Health Center (RHC) Business Plan of their health facility and its affiliated Health Houses (local health facilities). At the district level, the PHC management team, the main responsible body for initiating, monitoring and analyzing the Business Plans, elaborates a consolidated Business Plan of the health facilities of its district.

At the regional, district and national level, various user organizations participated in the scale-up process. The EPHC Services Project, international organizations, donor agencies and the Republican Center for Family Medicine (RCCFM) provide a wide range of technical, managerial and financial resources for scaling up. Table 1 shows the application of the elements of scaling up defined in the ExpandNet/WHO framework to the Business Plan scale-up process in Tajikistan.

#### Strategic choices regarding scaling up

**Figure 2.**
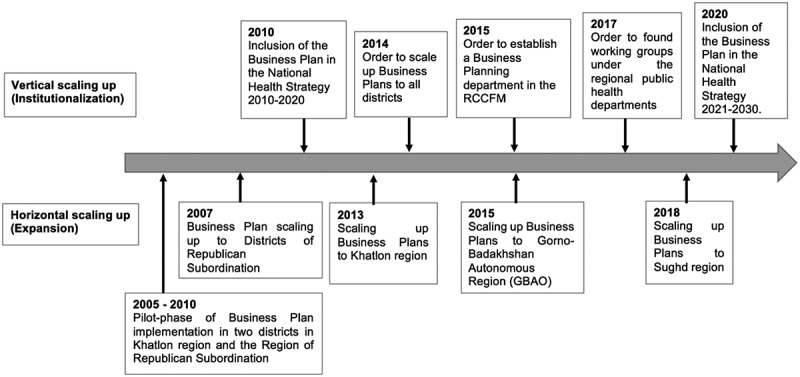
Milestones of the horizontal and vertical scaling up of Business Plans

The decision to scale up Business Plans was based on the positive feedback from the user organizations and evidence of the tool’s effectiveness, such as the improvement of health facility management skills, the increased transparency in the use of resources and the strengthened collaboration between health facilities and communities subsequently observed in the tailored choices of health priorities. The strategic choice areas for scaling up Business Plans are summarized in Table 2. A timeline of key events during Business Plan implementation and scaling up is shown in Figure 2.

**Table 2.** The strategic choice areas of scaling up Business Plans [12,21]

Strategic choice areas	Actions and decisions taken during Business Plan scaling up
**Type of scaling up**	Horizontal scaling up (expansion) Vertical scaling up (institutionalization)
**Organizational choices**	Participatory approach: MoHSP as the main decision-maker Guidance and advice by a working group on Business Plans consisting of leaders and specialists from the Department of Health Reform, PHC and International Relations, the Economics and Planning of Health Budgets department, the RCCFM, the Health Policy Analysis unit, representatives from the EPHC Services Project and representatives from the WHO Standardized implementation: Implementation of a uniform set of guidelines for the Business Plan introduction by the PHC management team Pace of scaling up: To our knowledge, no development of a clear long-term or multi-year scale-up strategy for guiding the process of going to full national scale with Business Plans Elaboration of annual plans with key activities within the Business Planning department and the regional working groups
**Cost and resource mobilization**	Assessment of costs: An investment case for the Business Plans is available. Provision of budget: Coverage of expenses regarding the expansion (horizontal scaling up) of Business Plans to new districts partly by the MoHSP and partly by external agencies, such as the Swiss Agency for Development and Cooperation (SDC), the Aga Khan Health Services and the WHO Budgetary allocation: Little change to the budget of PHC facilities during scaling up
**Monitoring and evaluation (M&E)**	Integration of M&E within the Business Plan At the district level, M&E through an Information Specialist in the PHC management team Discussion of the results from M&E directly with the implementers (heads of the rural healthcare facilities) and feedback via the consolidated Business Plan to the policy-makers (MoHSP) M&E of the scaling up process Jointly by the Business Planning department and the EPHC Services Project Additionally, through a steering committee consisting of representative of the government, the EPHC Services Project staff and the SDC
**Dissemination and Advocacy**	Policy dialogues: Advocacy for scaling up through champions from the RCCFM, the Department of Health Reform, PHC, and International Relations and other specialists within the ministry Networking and advocacy through the EPHC Services Project, for example in annual round tables with development partners, international organizations and potential donors Training: Conduction of training in a joint setting between the EPHC Services Project and the Business Planning department specialists Gradual transition of the full responsibility for national roll-out of Business Plans from the EPHC Services Project to the Business Planning department Implementation of cascade trainings between regional and district health departments and introduction of a management training module in the PGMI Training of Community Health Coordinators tasked with communicating the needs of the community with the PHC managers and the EPHC Services Project

### Facilitators

#### Facilitators related to the innovation

The integration of community health teams (CHTs) in the Business Plan elaboration is an important achievement throughout the scale-up process. A participant described that before the CHTs were included

the process was top-down. Now, the priorities are selected from the bottom up. The population’s knowledge and understanding of diseases has increased. The population itself chooses the necessary priorities […] This means they are free to choose their goals. [A, PHC management team]

Through the close cooperation with community members, heads of RHCs are able to tailor their priorities and activities set in the Business Plan to the specific needs of their community and to ensure that the Business Plan activities are relevant to the actual needs of the community.

Successful expansion of Business Plans (horizontal scaling up) also revolved around sufficient capacity and resources at the PHC facility level. Interviewees described that the key to success was having a well-trained management team at PHC and RHC facility level and receiving the guidelines and technical equipment to apply Business Plans.

#### Facilitators related to the user organization

Scaling up Business Plans was substantially facilitated by the support it received from the user organizations. Interviewees from the resource teams reported that the district PHC managers sent continuous positive feedback as Business Plans made facility management easier, motivated them to report data in a transparent way and allowed for more autonomy in budget execution. Interviewees at the ministerial level observed that PHC managers felt more responsible and accountable towards their communities.

#### Facilitators related to the resource team

From the perspective of the user organization, the technical expertise of the EPHC Services Project gained in the pilot phases facilitated the scale-up process. Interviewees emphasized that the long-term experience of the resource team was crucial to the successful introduction of Business Plans in the districts.

#### Facilitators related to the strategic choices during scaling up Business Plans

The institutionalization of the Business Plans in national orders of the MoHSP and their incorporation into national health policy documents as well as the introduction of per capita payment constituted a crucial achievement during scaling up. Taking a vertical scale-up approach by issuing orders and setting up the Business Planning department were seen as major milestones for the national roll-out of Business Plans:

So without the order, there would be no scaling up […] [H]ere something very formal has been established, there is a legal basis that gives a legal status to the Business Plan. It also creates structures and positions. [B, project staff]

Another important success was the inclusion of the Business Plans in the National Health Strategy (see Figure 2). Embedded in the political context, this form of institutionalization also gave the Business Plans importance and visibility, both for the donors and within the population. Scaling up Business Plans was further facilitated through advocacy by champions and policy-makers in the user organization, specifically the MoHSP, and the resource team.

With regard to dissemination, the large training network and the organization of continuous training opportunities have benefitted the roll-out of Business Plans. With the introduction of the cascade training approach, effective structures are in place to support the propagation of the tool. According to one interviewee, the effort put into training built a strong knowledge base:

There is kind of a critical mass of people that know about Business Planning and know how to use the Business Plan. […] So it’s not just like one or two people that know how to use a Business Plan, there is really plenty of people now that know how to use it, how to teach it. [C, project staff]

With regard to monitoring and evaluation, continuous communication between the central level (MoHSP) and the resource team (EPHC Services Project) facilitated the provision of information, feedback and organizational choices regarding the scale-up process.

### Barriers

#### Barriers related to the innovation

The complexity of the Business Plans is one of the main barriers to implementing Business Plans at a large scale because it requires both time-intensive, and from the national actors’ perspective cost-intensive, training of PHC managers. Another challenge within the scale-up process are potential alterations of the original objectives of the Business Plans. Some informants feared that scaling up could distort the intent of the Business Plan and that the tool could be used for collecting health-related data to inform national stakeholders:

It is also actually not meant for benchmarking because we know that the data has issues. […] It’s not meant to be a reporting tool and I think this is a risk when [the project] goes out, that people will use it as a reporting tool, which it is not. [B, project staff]

As described above, the Business Plan is designed to be a decentralized management tool used in healthcare facilities of the districts.

#### Barriers related to the user organization(s)

Constraints regarding scaling up Business Plans were overwhelmingly financial in nature, wherefore most participants emphasized that the capacity of the Business Planning department should be strengthened. Interviewees pointed out that financial resources are desperately needed to cover travel expenses for training purposes, for conducting monitoring and evaluation visits, to finance PHC managers’ trainings and to provide basic materials regarding Business Plans to health care facilities. Financial constraints were often also linked to the fact that the Business Planning department in the RCCFM is understaffed:

It is not possible for three people to train the whole country and then monitor and check and travel. It is really not possible for them to monitor this practice and implement [the Business Plans]. [D, project staff]

The lack of human resources was also closely associated with the lack of initiation and motivation to introduce Business Plans in new districts. Despite the resource team offering financial support for the first trainings of the healthcare workers and PHC managers (the most expensive part during the Business Plan introduction to a new district), the user organization remained hesitant to implement Business Plans. An interviewee pointed out that

one of the reasons is that there are not only financial limitations but that there is still a limitation in vision. That there is a limitation in the practical implementation of policy documents. [E, member of a donor agency]

At the district level, human resources were also identified by many informants as a problem area because the high staff turnover within the PHC management team can lead to a lack of institutional knowledge and additional costs. Similarly, some perceived the high staff turnover at the national level as a limitation to successfully scaling up Business Plans.

#### Barriers related to the strategic choices during Business Plan scaling up

Interviewees remarked that transferring responsibilities with respect to the implementation of Business Plans from the main resource team (the EPHC Services Project) to the user organization (RCCFM) should have focused on capacity building earlier in the scale-up process:

Because eventually you will anyhow have to do it. And the earlier the better. And you should invest your time more in checking them than doing groundwork (…). [B, project staff]

Some also pointed out that focusing more on strategic, organizational choices, such as the clarification of the functions and tasks of the new resource team or the pre-investigation of their implementation capacities, should have taken place in this transition process:

So we know that [the Business Planning department] is under-resourced but I don’t think we thought strategically enough [about] what exactly is our ambition and what we want this department to be able to do independently, by itself. And how we are going to monitor that it is getting there. Or how we are going to support it to get there. So, I think that that was not sufficiently well looked into. [C, project staff]

Interviewees remarked that the development of an overall scale-up strategy could have potentially mitigated the lack of human, material and financial resources, by having made these more explicit at an earlier stage. Others emphasized that not having a concrete or a practical multi-year scale-up plan posed a barrier to obtaining donor support.

#### Barriers related to the environment

In general, regional differences in the implementation capacity noticeable in the performance of the regional working groups seemed to be quite substantial. Interviewees suggested that these were mostly of financial or political nature. For example, due to pre-existing political difficulties, challenges arose during the expansion of Business Plans to Gorno-Badakhshan Autonomous Region (GBAO), the semi-autonomous region in Tajikistan. Besides political sensitivities, the remoteness of GBAO also posed limits to conduct trainings, and monitoring and evaluation in this area:

Access to GBAO is very difficult because in wintertime it is not possible to go there. Even if you can go there by car, you have to spend twelve or fourteen hours on the road. […] It is a very time and health forces consuming process. So, it is very difficult to monitor and help GBAO. [D, project staff]

Weaknesses within the health systems and health governance, such as a lack of vision, the incapacity to implement policies and a lack of medical education restricted scaling up Business Plans. The lack of far-reaching policy and structural reforms at PHC level, for example enabling independent access to budgets, has limited Business Plans in reaching their full scope, and can therefore also be seen as a barrier to scaling up. Further, missing intergovernmental collaborations, for example, with the finance departments or the local authorities (*hukumats*), also accounted for difficulties in scaling up, as important fiscal stakeholders were unaware of Business Plans.

A lack of effective donor coordination, the heterogeneity of interests among donors and the unclarity regarding the future of donor support for the tool emerged as challenges to sustainable scaling up. Not prioritizing primary health care, but focusing on the secondary and tertiary levels of care, both by the government and international agencies, diverted the attention away from financing the scaling up of the primarily PHC-oriented Business Plans.

## Discussion

### Overview of findings

The history of scaling up Business Plans is characterized by a complex multi-year consolidation process involving stakeholders at the national, district and regional level, and by vertical and horizontal scaling up approaches. The most prominent facilitator of scaling up was the vertical scale-up process, that is the institutionalization of Business Plans. Other determinants, such as the close collaboration with community members through CHTs, the high acceptance of Business Plans among the PHC managers, the technical expertise and experience of the user organization, the advocacy through champions and policy-makers and the large dissemination network for training and educating PHC-related staff on Business Plans enabled successful scale-up processes. The most outstanding barriers to scaling up Business Plans were insufficient financial or human resources for carrying out the implementation of Business Plans and weaknesses in the health governance system. Other aspects, such as, the lack of motivation to implement Business Plans, the difficulties in transitioning responsibilities and tasks between the user organization and the resource team, the lack of a strategic scale-up plan and the difficulties in donor coordination restricted scaling up Business Plans.

### Determinants and processes for successful scaling up

#### The importance of vertical scaling up

Our findings suggest that the institutionalization of the Business Plan through the establishment of its own department in the MoHSP was a milestone for ensuring its implementation on a larger scale. The importance of aligning an innovation with health policies and strategic health documents and embedding it in regulatory frameworks is highlighted in studies focusing on low- and middle-income countries [27,28] and in theoretical scaling up frameworks [12,21–23]. Another study using the ExpandNet/WHO framework for its analyses of scaling up efforts in Bangladesh has also highlighted the importance of institutionalization for successful scaling up, particularly with respect to logistics and distribution systems [29]. Especially in the context of Tajikistan’s historical legacy of vertical, centralized structures, the institutionalization of Business Plans is crucial. Mirroring our findings that the governmental adoption of Business Plans enables the large scale implementation of the tool, studies conducted in Ethiopia, India and Nigeria have emphasized that government ownership of an innovation can support scaling up because governments have the legitimacy and resources to foster this process [18,30]. Advocacy is another effective tool to involve governmental actors or policy champions and to influence decision-making [12,21,22,27]. However, Ministries of Health often face a lower political standing in the ministerial landscape and are structurally impeded in policy dialogues.

#### The importance of financial resources for successful scaling up

Financial resources are critical to scaling up [18]. The inequalities in the distribution of funds between administrative districts and regions, mainly favoring the capital Dushanbe and urban centers over rural peripheries [31], could explain the barriers to horizontally scaling up Business Plans, which were repeatedly described by the interviewees. To continue successfully scaling up Business Plans, not only the local authorities but also the Business Planning department need to be capacitated.

Often, donor funded projects in global health are limited in scale and have difficulties going to scale when donors exit the project [30,32]. In Tajikistan, where the health system is mainly vertically structured and a significant amount (8.22% in 2017) of total expenditure on health is provided by external resources [7,33], the exit of donors poses a serious challenge to sustain Business Plans in the future. To address the issues arising during the transition of donor-funded projects to local government-owned programs, enhancing donor coordination and building political commitment to replace donor-funding can ensure financial sustainability of innovative health programs [34,35]. In the case of scaling up Business Plans, both the EPHC Services Project and the MoHSP have taken action, for example, by organizing round tables with donors and supporting the WHO assessment of Business Plans, to ensure the credibility of the tool.

### Consolidation of lessons learnt

#### The importance of a scale-up strategy

As scaling up is a complex process requiring careful consideration of multiple factors, scaling up strategies are crucial to ensure that pilot-tested innovation can be *sustainably* implemented at a large scale [12,27] because they contain strategic choices regarding the allocation of resources, the dissemination, the training, the monitoring and the timing of scaling up [27]. Despite establishing the Business Planning department as a host institution for scaling up, insufficient attention has been directed towards increasing the capacity of the resource team in terms of financial or human resources. Taking into account that scaling up is usually conducted with limited resources and requires the integration of a new tool into the existing health system [12], developing a scale-up strategy for Business Plans could assess the feasibility and ensure the sustainability of the national roll-out.

#### The importance of systems thinking

Adhering to systems thinking [36] is one of the key guiding principles of the scale-up process [12]. A participatory process from testing the innovation to scaling it up can foster ownership of the innovation, especially when it spans all levels, ranging from community members to policy-makers [21]. Scaling up Business Plans has benefitted from the engagement of community members because their involvement greatly enhanced the sustainability of the tool and the effect of Business Plans on population health, especially compared to districts where CHTs were not involved [8]. Other studies support our finding that a strong network of community health workers facilitate the scale-up process, for example due to their involvement in the dissemination and application of health tools [28], their impact on local behavioral change [27] and their significance to understand and meet the needs of the communities [21]. Despite that scaling up usually follows a top-down approach [16], a process observable in the strongly vertically oriented scaling up of Business Plans, the Tajik government has taken steps towards engaging the public into scaling up Business Plans by legislating the involvement of CHTs in the implementation stage of the innovation, in particular the development of the Business Plans in PHC facilities. Taking into account that community-based PHC systems perform better [37], paying special attention to involving local CHTs is critical for scaling up Business Plans to the remaining districts.

The concept of systems thinking also emphasizes the analysis of the entire health system and the possibilities to strengthen its capacities. Structural changes, such as involving representatives of *hukumats* into scaling up Business Plans, was highlighted by several interviewees. As policy implementation and fund allocation to health facilities remain with district and regional authorities, the *hukumats* could facilitate the dissemination of Business Plan materials as well as support the implementation of the Business Plans in PHC facilities and the organization of CHTs.

### Strengths and limitations of the study

This study has included a large and diverse range of stakeholders from the community level up to the policy level. The interviews were conducted in the language the interviewees felt most comfortable to use, thus reducing bias caused by language barriers. Although we aimed to display a rich diversity of perspectives on the scale-up process, most interviewees held higher ranked policy positions and only the perspectives of management team representatives from two out of four district working groups and from one out of twenty-six districts could be represented. Therefore, there is a risk that some particular viewpoints may have dominated and others may have been missed. Synthesizing the data was further challenged by the lack of a uniform translation of the term ‘scale-up’ due to the abstractness and complexity of the term. Instead, other terms, such as ‘roll-out’, ‘expansion’ or ‘institutionalization’, were used to inquire about the processes and activities related to vertical and horizontal scale-up.

## Conclusion

The evaluation of scaling up Business Plans in Tajikistan once again shows that scaling up health innovations is a complex and multidimensional process. Successful scaling up is highly dependent on strategic decision-making and a careful assessment of the interrelations between the stakeholders at national, regional, district and local levels as well as the context and environment of the tool. To date, Business Plans are situated in a ‘hybrid scaled-up state’, having been subject both to scaling up on a horizontal level, as they have over time been expanded to cover almost half of Tajikistan’s health care facilities, and on a vertical level, as they have been institutionalized by the MoHSP. Considering that Business Plans have a positive impact on access to and provision of PHC services and that they have a strong base for their implementation on a nationwide scale, developing a scale-up strategy, strengthening cross-sectoral collaboration during scaling up and providing the user organizations of Business Plans with sufficient resources are important next steps to ensure the sustainability and effectiveness of Business Plans in the future.

## Figures and Tables

**Table:** 

**Scaling up process and management**	
***History and implementation of Business Plans***	
Please describe how the agreements and orders for the roll-out^1^ of Business Plans to the PHC facilities of the cities and *rayons* (districts) were reached. What steps were taken to establish the Business Planning department? How did the introduction of Business Plans affect the PHC facilities?	
***Questions specific to the interviewee sub-groups***	
Members of the MoHSP and the donor agencies	Now that there are formal orders and a fairly new department to manage the Business Plan roll-out^1^, how and by whom is the Business Plan roll-out^1^ to the PHC facilities organized?
Members of the EPHC Services Project	Who were your most important partners for making the national roll-out^1^ of Business Plans possible? Who were the most important people [politicians, policy-makers, institutions and organizations] to promote the roll-out^1^ of Business Plans to the PHC facilities of cities and *rayons* (districts)? Now that there are formal orders and a fairly new department to manage the Business Plan roll-out^1^, how and by whom is the Business Plan roll-out^1^ to the PHC facilities organized? Is it different to the past? Which responsibilities and activities related to the introduction of Business Plans in the PHC facilities could be transferred to the RCCFM so far?
Members of the regional Business Plan working groups	Now that there are formal orders and a fairly new department to manage the Business Plan roll-out^1^, please describe what your tasks are for the Business Plan roll-out to the PHC facilities. How did the introduction of Business Plans affect PHC facilities? Which responsibilities and activities related to the introduction of Business Plans in the PHC facilities have been transferred to you by the EPHC Services Project?
Members of the district level PHC management teams	Now that there are formal orders and a fairly new department to manage the Business Plan roll-out^1^, have you observed any differences in the way that Business Plans are introduced in the RHCs? Now that there are formal orders and a fairly new department to manage the Business Plan roll-out^1^, have you observed any differences in the way that Business Plans are used in the RHCs?
**Scaling up barriers and facilitators**	
From your experience, what has made extending/scaling-up/rolling-out Business Plans successful? What have been the major problems and challenges to extending/rolling-out Business Plans? How did you solve these problems? Looking back, what would you have done differently when rolling-out Business Plans?	
**Future scaling up**	
What challenges do you see in scaling up/rolling out Business Plans to the remaining districts? How could they be solved? What would your proposal be to make the Business Plan roll-out^1^ successful? What changes have to occur to ensure the use of the Business Plans in the future?	
**Wrap-Up**	
Would you like to add any remarks to help us understand your experience with the process of implementation and scale up better? Thank you very much for this interview and for your support of this study.	

^1^We used the term ‘roll-out’ to refer to scaling up in the interview questions because the stakeholders were more used to this terminology from prior studies and surveys conducted by the EPHC Services Project. Additionally, this term proved to be easier translated into Tajik and Russian.

## Paper context

This qualitative study was set in the frame of the Enhancing Primary Health Care Services Project in Tajikistan and adds knowledge to the field of scaling up by examining the process, particularly the enabling and restricting factors, of scaling up Business Plans (health facility management tools). Our findings suggest that developing a scale-up strategy, strengthening cross-sectoral collaboration and capacitating the tool’s user organizations are important next steps to ensure the sustainability of Business Plans.
